# Type II Heparin-Induced Thrombocytopenia Manifesting As Cardiac Arrest Following Intravenous Heparin Bolus During an Elective Procedure: A Case Report and Literature Review

**DOI:** 10.7759/cureus.57072

**Published:** 2024-03-27

**Authors:** Nisha Nepal, Dhiraj Patel, Opeyemi Omosebi, Yong Shin

**Affiliations:** 1 Internal Medicine, Danbury Hospital, Danbury, USA; 2 Internal Medicine, University of Vermont, Burlington, USA; 3 Internal Medicine, Connecticut Institute For Communities, Inc., Danbury, USA; 4 Medicine, Danbury Hospital, Danbury, USA

**Keywords:** anti-pf4 antibody, serotonin release assay, thromboembolism, pulmonary hemorrhage, cardiac arrest, direct anticoagulants, unfractionated heparin, anaphylactoid reaction, type ii hit

## Abstract

Heparin-induced thrombocytopenia (HIT) is a rare and life-threatening autoimmune-mediated adverse drug reaction seen in patients who are exposed to various forms of pharmacological heparin, including unfractionated heparin (UFH) and low molecular weight heparin (LMWH). Despite the presence of thrombocytopenia, these patients face the risk of clot formation and bleeding simultaneously. Prompt cessation of heparin and the initiation of non-heparin anticoagulants are important for the patient's survival. Typically, clinical diagnosis of HIT is necessary, and waiting for lab test results, which can take days, may not be always feasible. Here, we present a case of an unusual presentation of type II HIT, complicated by significant thrombocytopenia, pulmonary hemorrhage, and cardiac arrest after receiving intravenous (IV) heparin bolus during an elective cardiac ablation procedure for paroxysmal atrial fibrillation.

## Introduction

The pathophysiology of heparin-induced thrombocytopenia (HIT) involves auto-antibodies, most commonly immunoglobulin G, targeting molecular complexes of platelet factor 4 (PF4) and heparin (PF4-heparin complexes) [1,2]. As these complexes, also referred to as HIT antibodies, circulate within the bloodstream, they bind and activate platelets leading to thrombocytopenia and thrombosis due to aggregation of activated platelets. Despite the formation of thrombocytopenia, the activation of platelets and the formation of thrombi lead to a hypercoagulable state. Two forms of HIT exist: type I HIT is more common and is a non-immune mediated disease that presents 1-3 days after heparin exposure with mild and transient thrombocytopenia. On the other hand, type II HIT, an autoimmune-mediated condition, occurs 5-14 days after exposure and is more clinically significant as it carries a greater risk for thromboembolic events compared to type I HIT [3]. A subset of type II HIT, rapid onset HIT, found in 25% of cases, involves the development of thrombocytopenia within 24 hours of re-exposure due to circulating antibodies from recent heparin use within the last 90 days. It is usually complicated by an anaphylactoid reaction during the 30 minutes after a heparin bolus [4]. Delay in treatment of HIT may result in a 5-10% daily risk of thrombosis, amputation, or death [5]. When suspecting HIT, an initial step is to assess the 4T score, encompassing thrombocytopenia, timing of platelet fall, thrombosis, and other causes of thrombocytopenia. With scores ranging from 0 to 8, a low probability 4T score (<=3) demonstrates an impressive negative predictive value of almost 99%. However, patients falling into the intermediate probability range (4T score 4 or 5) or the high probability category (>=6) necessitate further tests like immunoassay and serotonin release assay (SRA) to rule in or rule out the diagnosis [6,7]. The first test is to perform an immunoassay which if negative rules out the diagnosis but if positive it needs to be confirmed with SRA. If the SRA is negative then HIT is ruled out but positive SRA is diagnostic of HIT. In the management of acute HIT, the critical initial step is the prompt discontinuation of heparin. Simultaneously, intervention with non-heparin products becomes imperative. Options include argatroban, bivalirudin, danaparoid, fondaparinux, or a direct oral anticoagulant (DOAC) [8]. Warfarin should be avoided in patients with HIT until the platelets become normalized [9]. Here, we discuss an unusual presentation of type II HIT complicated by significant thrombocytopenia, pulmonary hemorrhage, and cardiac arrest while undergoing an elective cardiac ablation procedure for paroxysmal atrial fibrillation (pAF).

## Case presentation

The patient, a woman in her 70s, presented for cryoablation for pAF. Unfortunately, the patient went into pulseless electrical activity (PEA) while undergoing the procedure shortly after receiving intravenous (IV) heparin bolus 6000 units. She has a medical history spanning two and a half years, including pAF managed with chronic anticoagulation using apixaban. Additionally, she has a history of congestive heart failure, hypertension, hyperlipidemia, hypercholesterolemia, osteopenia, depression, anxiety, and gastroesophageal reflux disease.

While dealing with pAF, the patient faced challenges in achieving consistent rate control with various anti-arrhythmic medications. Hospitalization was required on multiple occasions to address her rapid heart rate from AF. In the same year, the patient underwent cholecystectomy for acute cholecystitis, leading to the temporary discontinuation of apixaban during the peri-operative period. She was managed with the heparin drip during this period, but on the day of surgery, it was stopped. She was discharged a day later with advice to resume the anticoagulant. Unfortunately, during the period without anticoagulation, she had a cerebrovascular accident (CVA), the CT scan head showed a left frontal lobe infarct at that time. While recovering from the CVA, the patient resumed her regular dose of apixaban and metoprolol and was started on diltiazem for rate control. Approximately six weeks later, she presented for elective atrioventricular (AV) nodal ablation. Just prior to the procedure, she received 6000 units of IV heparin. The procedure started with cardioversion and cryoablation was commenced on the left superior pulmonary vein. However, she went into acute hypoxemic and hypercapnic respiratory failure followed by PEA cardiac arrest. Cardiopulmonary resuscitation (CPR) was started and return of spontaneous circulation (ROSC) was achieved after six minutes. Blood was seen from the endotracheal tube, but the bleeding stopped after protamine administration. Emergent bronchoscopy revealed spontaneous pulmonary hemorrhage in the right lower lobe, which was successfully stopped with iced saline washes.

A chest CT angiogram ruled out pulmonary embolism, and CT scans of the brain, abdomen, and pelvis showed no acute abnormalities. Due to the pulmonary hemorrhage leading to cardiac arrest, apixaban was withheld. However, after extubation the following day when there were no signs of bleeding, anticoagulation was resumed immediately given her recent CVA a few weeks ago when the anticoagulation was stopped for a cholecystectomy procedure. Heparin drip was used instead of apixaban due to its shorter half-life compared to apixaban, considering the recent pulmonary hemorrhage during the ablation procedure.

In the next few days, the patient's platelets dropped significantly from 215x10^9/L (reference range: 150x10^9/L-400x10^9/L) one month prior to 83x10^9/L and then 36x10^9/L and thus HIT type II was suspected. Our patient had a HIT score of 7, with criteria including a platelet count fall of >50% AND a platelet nadir of >=20, which contributed 2 points. Additionally, a fall <1 with prior heparin exposure 30-100 days ago gave 1 point, an acute systemic reaction post-heparin bolus contributed 2 points, and the absence of apparent causes of thrombocytopenia yielded 2 points, resulting in a total score of 7, indicating a high probability of HIT. Hence, the heparin drip was discontinued, and an argatroban drip was started. Various tests were done to rule out other potential causes of thrombocytopenia like disseminated intravascular coagulation (DIC) and hemolytic anemia. The peripheral smear revealed no morphologic abnormality of platelets, and red blood cells appeared normocytic normochromic, with some exhibiting basophilic stippling. Her fibrinogen levels were elevated at 534 mg/dl (reference range: 210-480 mg/dl), which ruled out DIC. The international normalized ratio (INR) was found to be mildly high at 15.1 seconds (normal range: 12.2-14.5 seconds), and lactate dehydrogenase (LDH) levels were elevated at 308 U/L (normal range: 122-222 U/L). Direct antiglobulin test (DAT) and liver function tests, including bilirubin and transaminases, showed normal results ruling out hemolysis (Table 1).

**Table 1:** Pertinent lab test results INR: international normalized ratio, LDH: lactate dehydrogenase, AST: aspartate aminotransferase, ALT: alanine aminotransferase

Test	Result	Normal Range
Fibrinogen	534 mg/dl	210-480 mg/dl
INR	15.1 seconds	12.2-14.5 seconds
LDH	308 U/L	122-222 U/L
Bilirubin	1.1 mg/dl	0-1.2 mg/dl
Transaminases (AST and ALT)	AST 32 U/L; ALT 43 U/L	AST (10-50 U/L); ALT (10-55 U/L)

**Figure 1:**
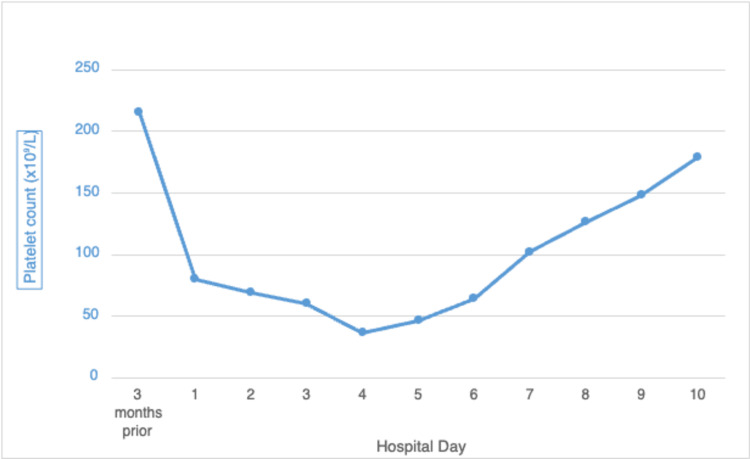
The patient's platelet count during her hospitalization Her baseline platelets three months prior were 215x10^9/L (reference range: 150-400x10^9/L), on her hospital day 1 right after the anaphylactoid reaction the platelets were 80x10^9/L. On hospital day 3, a heparin drip was started to avoid cerebrovascular accident (CVA) in the background of her atrial fibrillation. On her hospital day 4, when her platelets dropped to 36x10^9/L, heparin-induced thrombocytopenia (HIT) type II was suspected, the heparin drip was stopped and the agratobran drip was started. Subsequently, on her hospital day 7, her platelets increased to 126x10^9/L. At that time agratobran was stopped and apixaban was started. She was discharged home on hospital day 9 when her platelets were 179x10^9/L.

The DAT was negative. HIT PF4 antibodies returned positive, and an SRA also came back positive after a few days. After the normalization of the platelet count, apixaban was resumed without any side effects, and the patient was discharged home. The summary of her platelet count and the timing of heparin discontinuation along with non-heparin anticoagulant administration until the discharge is depicted in Figure 1 as shown below.

## Discussion

Our patient who has a past medical history of AF, who had been on a heparin drip a few weeks ago before undergoing a surgical procedure experienced adverse reactions including cardiac arrest to IV heparin bolus six weeks later during elective AV nodal ablation. After successful resuscitation, she was started on a heparin drip to avoid a CVA which she experienced in the recent past while being off of anticoagulants. HIT type II was suspected only a day later when her platelet count dropped more than 50% of her baseline. In retrospect, the cardiac arrest after receiving IV heparin bolus was the anaphylactoid reaction in type II HIT. The heparin drip was stopped, and she was started on an argatroban drip. Although the exact number of case reports of anaphylactoid reaction to heparin is not known, there are eight case reports regarding IV heparin bolus-induced anaphylactoid reaction found in the literature [10]. Because of the low incidence of anaphylactoid reaction, HIT type II isn’t always considered in the very beginning until the platelets start dropping significantly like in our case. Furthermore, through our extensive literature review, this case is the first reported case of a patient with type II HIT where the patient, fortunately, didn’t have any other clinical complications apart from thrombocytopenia while being on the heparin drip for a few hours. It is possible that the anaphylactoid reaction resulting in cardiac arrest was due to the high dosage of IV heparin (e.g., 6000 units IV at once), and with the drip, there is a much lower dosage administration (e.g., around 350 units in 24 hours). However, because of the extremely low number of cases reported, there is a limitation regarding this hypothesis.

## Conclusions

In conclusion, this case emphasizes the need for heightened awareness among healthcare providers regarding some of the complexities in recognizing HIT. The patient's presentation of cardiac arrest following an IV heparin bolus during an elective procedure underscored the importance of considering HIT type II in such scenarios, particularly when there is a significant rapid drop in platelet count. Timely recognition and appropriate management, including discontinuation of heparin and initiation of alternative anticoagulation such as argatroban, played a crucial role in mitigating further complications.
